# Definition der Hüftdysplasie im Jahr 2023: Zeichen der Makro- und Mikroinstabilität

**DOI:** 10.1007/s00132-023-04353-x

**Published:** 2023-03-07

**Authors:** Alexander Frank Heimann, Corinne Andrea Zurmühle, Vera Marie Stetzelberger, Julien Galley, Joseph M. Schwab, Moritz Tannast

**Affiliations:** 1 Klinik für Orthopädische Chirurgie und Traumatologie, HFR Kantonsspital Freiburg, Universität Freiburg, Chemin des pensionnats 2–6, 1700 Freiburg, Schweiz; 2 Klinik für Radiologie, HFR Kantonsspital Freiburg, Universität Freiburg, Freiburg, Schweiz

**Keywords:** Hüftgelenk, Entwicklungsbedingte Hüftdysplasie, Femoroazetabuläres Impingement, Hüftluxation, Periazetabuläre Osteotomie, Acetabulofemoral joint, Developmental dysplasia of the hip, Femoracetabular impingement, Hip dislocation, Periacetabular osteotomy

## Abstract

**Hintergrund:**

Die Hüftdysplasie ist eine komplexe statisch-dynamische Pathologie, welche zu chronischer Gelenkinstabilität und Arthrose führt. Das Verständnis der zugrundeliegenden Pathomorphologie hat sich weiterentwickelt, sodass eine Aktualisierung der Definition erforderlich ist.

**Fragestellung:**

Wie lautet die Definition der Hüftdysplasie im Jahr 2023?

**Methoden:**

Durch Zusammenfassung und Aufarbeitung der relevanten Literatur wird eine aktuelle Definition der Hüftdysplasie mit konkreten Angaben zur Diagnostik bereitgestellt.

**Ergebnisse:**

Neben pathognomonischen sind supportive und deskriptive Parameter, sowie sekundäre Veränderungen von zentraler Bedeutung zur Diagnose der Hüftinstabilität. Die diagnostische Basis ist die konventionelle Beckenübersichtsaufnahme, welche bei Bedarf durch Zusatzuntersuchungen (Arthro-MRT der Hüfte; CT) ergänzt wird.

**Schlussfolgerung:**

Die Komplexität, Subtilität und Vielfalt der Pathomorphologie residueller Hüftdysplasien erfordert eine sorgfältige, mehrstufige Diagnostik und Therapieplanung in spezialisierten Zentren.

## Abstract (en)

**Background:**

Hip dysplasia is a complex static-dynamic pathology leading to chronic joint instability and osteoarthritis. Because our understanding of the underlying pathomorphologies of hip dysplasia, both on the macro and micro levels, has evolved, an updated definition is needed.

**Question:**

What is the definition of hip dysplasia in 2023?

**Methods:**

By summarizing and reviewing relevant literature, we provide an up-to-date definition of hip dysplasia with a guide to appropriately making the diagnosis.

**Results:**

In addition to the pathognomonic parameters, supportive and descriptive indicators, as well as secondary changes are used to fully characterize instability inherent in hip dysplasia. The primary diagnostic tool is always the plain anteroposterior pelvis radiograph, which can be supplemented by additional investigations (MRI of the hip with intraarticular contrast agent; CT) if necessary.

**Conclusion:**

The complexity, subtlety, and diversity of the pathomorphology of residual hip dysplasia requires careful, multilevel diagnosis and treatment planning in specialized centers.

Die klassische Definition der Hüftdysplasie aus dem Jahr 1939 wurde über ein halbes Jahrhundert unverändert gebraucht. Die früher häufig gesehenen hohen Luxationen sind seit der Einführung der Screening-Untersuchung durch residuelle, subtilere Pathologien abgelöst worden. Zusätzlich hat der immense Fortschritt in Bildgebung und chirurgischen Therapiemöglichkeiten der letzten 20–30 Jahre neue pathomechanische Erkenntnisse ermöglicht, wodurch es einem Update über die Definition und Diagnostik der Hüftdysplasie und einer Diskussion über Makro- und Mikroinstabilitäten bedarf.

## Ätiologie

Die genaue Ätiologie der Hüftdysplasie ist bis heute nicht bekannt. Wir gehen von einer multifaktoriellen Genese durch hormonelle, genetische und konstitutionelle Faktoren aus. Dazu zählen neben einer positiven Familienanamnese und weiblichem Geschlecht eine Beckenendlage während der Schwangerschaft [6], oder anderweitig bedingter intrauteriner Platzmangel, wie er bei einer Zwillingsschwangerschaft oder einem Oligohydramnion [14] vorkommt. Ursächlich dabei ist vermutlich eine Dezentrierung des Femurkopfes, welche zu einer erhöhten Druckbelastung am Pfannenerker mit konsekutiver Unterentwicklung der Hüftgelenkspfanne führt. Eine Hüftdysplasie kann des Weiteren auch mit anderen Fehl- oder Missbildungen wie der infantilen Zerebralparese, Klump‑/Knick-Senk-Füssen oder dem kongenitalen Tortikollis assoziiert sein, oder, beispielsweise im Falle eines Morbus Perthes, nach Trauma oder einer septischen Arthritis sekundär entstehen.

Grundsätzlich entwickelt sich das ätiologisch-pathomechanische Verständnis der Hüftdysplasie weg von einer isolierten Betrachtung der lateralen Überdachung des Femurkopfes hin zu einer Betrachtung der Dysplasie als komplexe, statisch-dynamische Pathomorphologie mit eventuell sogar syndromartigem Charakter. So konnten Unterschiede in der Gesichtsmorphologie von Dysplasiepatienten im Vergleich zur Normalbevölkerung festgestellt werden [5]. Bleibt eine kongenitale oder im Kindesalter sekundär entstandene Hüftdysplasie unbehandelt oder unerkannt und führt erst im Erwachsenenalter zu klinischen Beschwerden, spricht man von einer residuellen Hüftdysplasie. Diese häufig subtileren Pathomorphologien, die bis ins Erwachsenenalter fortbestehen, gewinnen immer mehr an Bedeutung. Denn auch sie führen zu chronischer Gelenkinstabilität und, wenn sie unbehandelt bleiben, zu frühzeitiger Koxarthrose.

## Historische Definition der Hüftdysplasie

Die ursprüngliche Definition der Hüftdysplasie basiert auf der Vorstellung, dass es aufgrund einer azetabulären Fehlentwicklung zu einer steiler geneigten, lateral defizitären Hüftgelenkspfanne mit verminderter Überdachung des Femurkopfes kommt. Diagnostisch wird die laterale Überdachung des Hüftkopfes seit 1939 mittels lateralem Zentrum-Erker-Winkel (englisch „LCE angle“) auf einer konventionellen Beckenübersichtsaufnahme im a.-p. Strahlengang nach Wiberg beurteilt [31].

Im Wandel der Zeit – von Dysplasie zu Instabilität

Weitere Winkel wie der azetabuläre Winkel nach Sharp zur Beschreibung der mangelhaften lateralen Überdachung [19], oder der vertikale Zentrum-Eck-Winkel [10] zur Quantifizierung der anterioren Überdachung schufen ein zunehmend dreidimensionales Bewusstsein der Pathomorphologie. Dadurch kamen in den 1990er-Jahren viele weitere radiologische Parameter hinzu, mit deren Hilfe subtilere Morphologien erkannt und beschrieben werden können. Und obwohl zwischenzeitlich mit der Computertomographie (CT) oder der Magnetresonanztomographie (MRT) modernere Techniken zur Verfügung stehen, bildet die klinische Untersuchung in Verbindung mit der klassischen Röntgendiagnostik nach wie vor die Basis der Hüftdysplasiediagnostik. Heute steht nicht mehr isoliert die laterale Überdachung, sondern eine Bewertung der Instabilität im Mittelpunkt.

## Pathomechanismus der Hüftdysplasie

Folgende zwei Pathomechanismen führen in dysplastischen Hüften zur Gelenkinstabilität:

- ein defizitäres Containment des Hüftkopfes
- die Inkongruenz der artikulierenden Gelenkflächen [21]

Die Facies lunata ist in dysplastischen Hüften verkleinert [21]. Das resultierende Containment-Defizit des Femurkopfes kann hierbei je nach Orientierung der Hüftpfanne anterolateral, lateral oder posterior vorliegen [13]. Durch die verkleinerte lastübertragende Kontaktfläche zwischen Azetabulum und proximalem Femur kommt es zu einer statischen mechanischen Überbelastung der betroffenen Hüftgelenke. Azetabulär kommt es dadurch zu sekundären Veränderungen des Pfannendachrandes, während es femoral zu einer progredienten Verformung des Hüftkopfes mit konsekutiver Subluxation kommt [2]. Es entwickelt sich ein zunehmend inkongruentes Gelenk, bei dem die Drehzentren von Hüftkopf und -pfanne nicht mehr übereinstimmen. Dies trägt maßgeblich zur resultierenden chronischen Instabilität dysplastischer Hüftgelenke bei.

Im Verlauf kommt es auf dem Boden dieser Pathomorphologie zu Schmerzen und der frühzeitigen Entwicklung einer Koxarthrose als direkte Folge des Überdachungsdefizites [15, 30]. Nach heutigen Schätzungen ist die Hüftdysplasie, nach dem femoroazetabulären Impingement, mit einer Prävalenz von bis zu 40 % vermutlich die zweithäufigste Ursache einer Koxarthrose [3].

## Klinische Symptomatik

Das klinische Beschwerdebild kann als symptomatisch werdende Instabilität verstanden werden, überschneidet sich jedoch maßgeblich mit anderen Hüftpathologien, wie dem femoroazetabulären Impingement-Syndrom. Insbesondere der Schmerzkontext kann jedoch bei der Unterscheidung von Instabilität und Impingement hilfreich sein: So zeigen sich durch eine Instabilität verursachte Schmerzen typischerweise während oder nach der Belastung häufig am lateralen Hüftbereich, beziehungsweise langem Stehen und/oder Treppab-Gehen. Als Abgrenzung hierzu zeigen sich typische Impingement-Schmerzen in der Leiste eher bei langem Sitzen oder repetitiven Flexions- und Innenrotationsbewegungen. Ergänzend können Begleiterscheinungen wie ein vernehmbares Schnappen oder eine Hypermobilität vorliegen.

**Figure 1:**
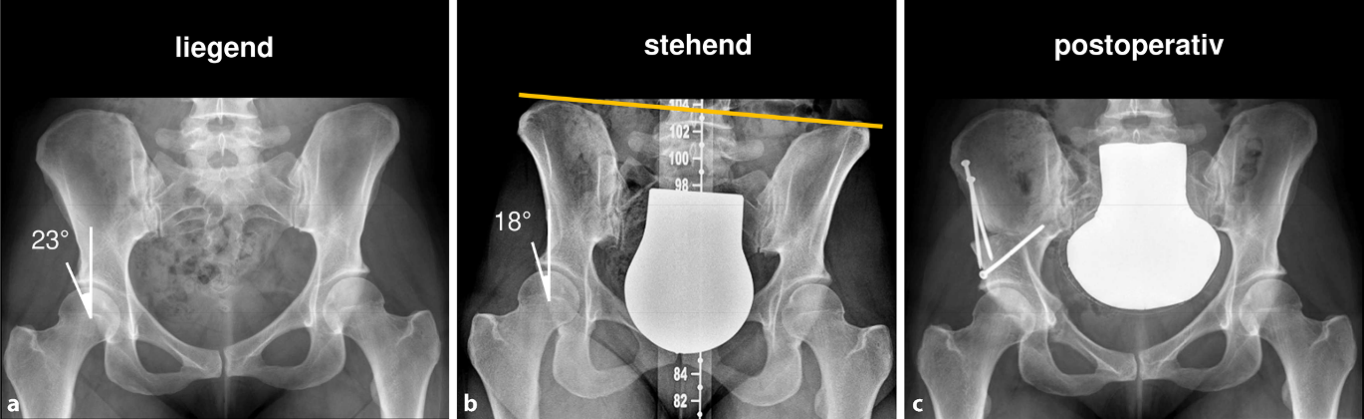

Ziel der körperlichen Untersuchung ist das dynamische, reproduzierbare Auslösen der typischen Beschwerden durch den Untersucher. Dies erlaubt einen indirekten Rückschluss auf die zugrundeliegende Schmerzursache. Nach Überprüfung des Bewegungsumfangs und der klassischen Schmerzprovokationstests (Flexion-Adduktion-Innenrotation und Flexion-Abduktion-Außenrotation) im Seitenvergleich sollte das Augenmerk auch auf eine mögliche Beinlängendifferenz (Abb. 1) oder femorale Torsionsfehler gerichtet werden.

## Radiologische Beurteilung

**Figure 2:**
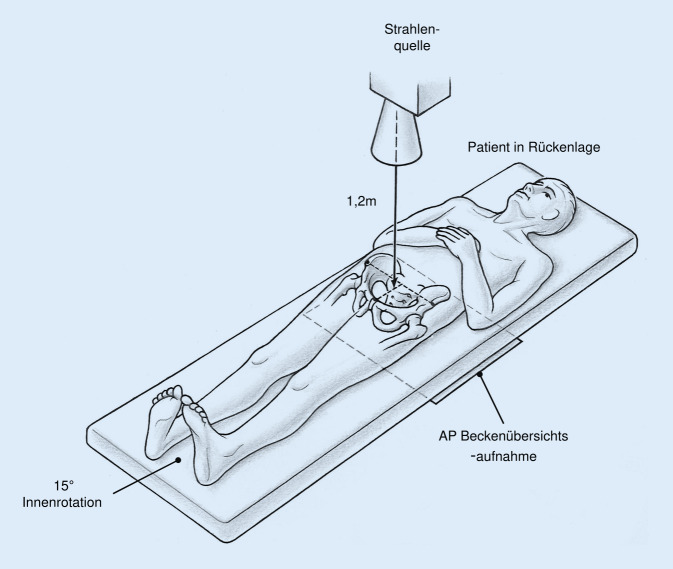

Den Goldstandard zur Diagnose der Hüftdysplasie stellt nach wie vor die konventionelle a.-p. Beckenübersichtsaufnahme dar. Hierbei gilt es zu beachten, dass eine standardisierte, beckenzentrierte Aufnahmetechnik (Abb. 2) zur Anwendbarkeit und Vergleichbarkeit der radiologischen Parameter zwingend erforderlich ist, da ansonsten eine relevante Fehlinterpretation insbesondere der azetabulären Vorder- und Hinterwand möglich ist [27].

### Pathognomonische, supportive und deskriptive Parameter

**Figure 3:**
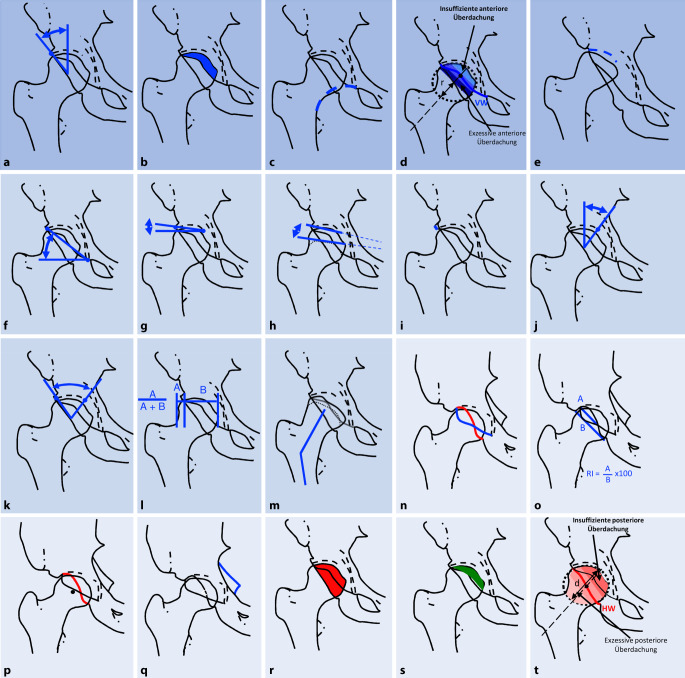

Eine Übersicht der konventionell-radiologischen Parameter zur Beurteilung der Hüftdysplasie anhand einer a.-p. Röntgenaufnahme des Beckens ist in Abb. 3 und Tab. 1 dargestellt. In Tab. 2 sind die zugehörigen Referenzwerte angegeben.

**Table 1:** 

Parameter	Definition	Abb. 3
*Pathognomonische Parameter*	Alleinig zur Diagnose der Hüftgelenksdysplasie ausreichend	Abb. 3a–e
„Lateral-center-edge“(LCE)-Winkel (°) [31]	Winkel zwischen Gerade durch den lateralen Pfannenrand und das Femurkopfzentrum und der Vertikalen	Abb. 3a
Anteriore Überdachung (%) [26]	Anteil des Hüftkopfes, der vom vorderen Pfannenrand in a.-p. Richtung bedeckt ist	Abb. 3b
Shenton-Linie [7]	Linie entlang des Schenkelhalsunterrandes, die harmonisch in den Unterrand des Ramus superior ossis pubis übergeht	Abb. 3c
Drittel-Regel azetabuläre Vorderwand [23]	Schnittpunkt des vorderen Pfannenrandes mit der Schenkelhalsachse im Verhältnis zum Hüftkopfradius	Abb. 3d
Hohe Luxation	Proximalisierter Hüftkopf mit Bildung eines Neoazetabulums am proximalen Pfannenpol	Abb. 3e
*Supportive Parameter*		Abb. 3f–n
Sharp Winkel (°) [19]	Winkel, der durch die Horizontale und eine Gerade durch das kaudale Ende der Köhler-Tränenfigur und den lateralen Pfannenrand gebildet wird	Abb. 3f
Azetabulärer Index (°) [29]	Winkel, der durch die Horizontale und eine Gerade durch den medialsten Punkt der Sklerosezone und den lateralen Pfannenrand gebildet wird	Abb. 3g
Femoroepiphysealer azetabulärer „Roof“-Index (FEAR-Index, °)	Winkel zwischen einer Geraden durch den medialsten Punkt der Sklerosezone und den lateralen Pfannenrand und einer Geraden entlang des mittleren Drittels der Epiphysennarbe des Hüftkopfes	Abb. 3h
Lateral aufsteigende Braue	Neigung des lateralen Pfannenrandes von kaudal nach kranial mit Verlust der normalen lateralen Konkavität des Azetabulums	Abb. 3i
„Medial-center-edge“(MCE)-Winkel (°) [25]	Winkel, der durch eine zur Beckenlängsachse parallele Linie und eine Verbindungslinie zwischen der Mitte des Hüftkopfes mit dem medialen Rand der Hüftgelenkspfanne gebildet wird	Abb. 3j
Azetabulärer Bogen (°) [25]	Summe aus LCE- und MCE-Winkel. Winkel, der durch zwei Linien gebildet wird, die durch das Zentrum des Hüftkopfes und den medialen, beziehungsweise lateralen Rand der Hüftgelenkspfanne gehen	Abb. 3k
Femurkopfextrusionsindex (%) [15]	Prozentualer Anteil des unbedeckten Hüftkopfes (*A*) im Vergleich zum gesamten horizontalen Hüftkopfdurchmesser (*A* *+* *B*)	Abb. 3l
Femurkopfform [22]	Form des Hüftkopfes; bei Dysplasie typischerweise abgeflacht/entrundet, mit hoher Fovea capitis femoris, schmaler Epiphyse und ggf. valgischer Konfiguration des Schenkelhalses	Abb. 3m
Fovea alta [17]	Kranialisierte Fovea capitis femoris, die in die Hauptbelastungszone hineinreicht	Abb. 3m
*Deskriptive Parameter*		Abb. 3n–t
Überkreuzungszeichen („cross-over sign“) [20]	Positiv, wenn die projizierte azetabuläre Vorderwand die Hinterwand kreuzt	Abb. 3n
Retroversionsindex (%) [20]	Der prozentuale Anteil der Überlappung des anterioren und posterioren Pfannenrandes im Vergleich zur gesamten Länge der lateralen Azetabulumöffnung	Abb. 3o
Hinterwandzeichen („posterior wall sign“) [24]	Positiv, wenn der hintere Pfannenrand medial des Hüftkopfzentrums liegt	Abb. 3p
Spina-ischiadica-Zeichen („ischial-spine sign“) [8]	Positiv, wenn die Spina ischiadica innerhalb des Beckenrings sichtbar ist	Abb. 3q
Posteriore Überdachung (%) [26]	Prozentualer Anteil des Hüftkopfes, der vom vorderen Hüftpfannenrand in a.-p. Richtung bedeckt ist	Abb. 3r
Kraniokaudale Überdachung (%) [26]	Prozentualer Anteil des Hüftkopfes, der von der Hüftpfanne in kraniokaudaler Richtung bedeckt ist	Abb. 3s
Drittel-Regel azetabuläre Hinterwand [23]	Schnittpunkt des hinteren Pfannenrandes mit der Schenkelhalsachse im Verhältnis zum Hüftkopfdurchmesser	Abb. 3t

**Table 2:** 

Parameter	Dysplasie	Normal	Pincer
*Pathognomonische Parameter*			
„Lateral-center-edge“(LCE)-Winkel (°) [31]	< 22	23–33	34–39
Anteriore Überdachung (%) [26]	< 14	15–26	27–32
Shenton-Linie [7]	Unterbrochen	Intakt	Intakt
Drittel-Regel azetabuläre Vorderwand [23]	VW-SHA-SP im ant./mittl./post. Drittel	VW-SHA-SP im mittl. 1/3	VW-SHA-SP im ant./mittl./post. Drittel
Hohe Luxation	+/−	−	−
*Supportive Parameter*			
Sharp-Winkel (°) [19]	> 43	38–42	34–37
Azetabulärer Index (°) [29]	> 14	3–13	−7–2
Femoroepiphysealer azetabulärer „Roof“-Index (FEAR-Index, °)	> 5	< 5	n. a.
Lateral aufsteigende Braue	+/−	−	−
„Medial-center-edge“(MCE)-Winkel (°) [25]	> 45	35–44	34–29
Azetabulärer Bogen (°) [25]	< 60	61–65	66–69
Femurkopfextrusionsindex (%) [15]	> 27	17–27	12–16
Femurkopfform [22]	Elliptisch	Sphärisch	Sphärisch
Fovea alta [17]	+/−	−	−
*Deskriptive Parameter*			
Überkreuzungszeichen („cross-over sign“) [20]	+/−	−	−
Retroversionsindex (%) [20]	≥ 40	< 40	k. A.
Hinterwandzeichen („posterior wall sign“) [24]	+/−	+	+/−
Spina-ischiadica-Zeichen („ischial-spine sign“) [8]	+/−	−	−
Posteriore Überdachung (%) [26]	< 35	36–47	48–55
Kraniokaudale Überdachung (%) [26]	< 69	70–83	84–93
Drittel-Regel azetabuläre Hinterwand [23]	HW-SHA-SP im ant./mittl./post. Drittel	HW-SHA-SP im mittl. Drittel	HW-SHA-SP im ant./mittl./post. Drittel

*VW-SHA-SP* Vorderwand-Schenkelhalsachse-Schnittpunkt, *HW-SHA-SP* Hinterwand-Schenkelhalsachse-Schnittpunkt

+ positiv, − negativ

Es existieren fünf pathognomonische Parameter, deren alleiniges Vorliegen im dysplastischen Referenzwertbereich zur Diagnose einer Hüftdysplasie ausreichend ist. Hierzu zählen ein LCE-Winkel < 22° [25], eine anteriore Hüftkopfüberdachung < 14 % [25], eine unterbrochene Shenton-Linie [7], eine insuffiziente anteriore Überdachung nach der Drittel-Regel für die azetabuläre Vorderwand [23] sowie eine hohe Luxation (Abb. 3a–e). Typischerweise handelt es sich in diesen Fällen um ausgeprägte Instabilitäten.

Zum Nachweis der Instabilität in Grenzfällen werden supportive Parameter in Verbindung mit der klinischen Symptomatik (Abb. 3f–m) benötigt, um die Diagnose einer Hüftdysplasie stellen zu können. Insbesondere bei den sogenannten Borderline-Dysplasien (historisch definiert als LCE-Winkel von 20–25° [31]) können auch Überschneidungen mit einem femoroazetabulären Impingement vorliegen.

Deskriptive Parameter (Abb. 3n–t) sind zur dreidimensionalen morphologischen Analyse hilfreich, um im Einzelfall die Ursache der Hüftschmerzen in pathomorphologisch komplexen Fällen der entsprechenden Hüftpathologie zuzuordnen und um nachfolgend die geeignetste chirurgische Korrektur zu definieren, beispielsweise die Art der Reorientierung bei einer azetabulären Umstellungsosteotomie.

Die Diagnose der Hüftdysplasie ist heutzutage meist nicht mehr auf einen einzigen radiologischen Parameter zurückzuführen, sondern ergibt sich als Summe verschiedener Parameter in Zusammenschau mit der klinischen Untersuchung.

### Messung der femoralen Torsion

**Figure 4:**
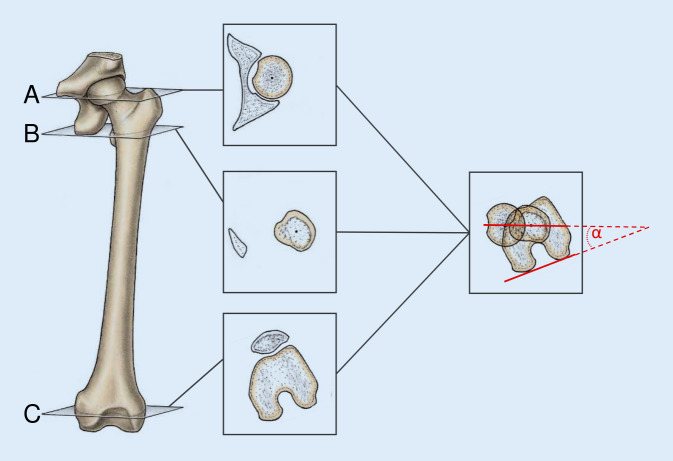

Fast 90 % der dysplastischen Hüften weisen eine pathologische femorale Torsion auf, wobei tendenziell häufiger eine erhöhte femorale Antetorsion vorliegt [11, 12]. Da eine pathologisch erhöhte (> 25° nach Murphy) oder verminderte (< 10° nach Murphy) femorale Antetorsion das Zusammenspiel von Hüftkopf und -pfanne unmittelbar beeinflusst und somit ein entscheidender Faktor der Gelenkstabilität ist, empfehlen wir grundsätzlich die Durchführung einer Torsionsmessung mittels CT oder MRT. Die Messmethode ist hierbei immer mit dem Messwert zu nennen, da sich die Messwerte der unterschiedlichen beschriebenen Messmethoden erheblich voneinander unterscheiden können [18]. Im klinischen Alltag hat sich bei uns die Messmethode nach Murphy bewährt ([16]; Abb. 4). In den letzten Jahren hat sich zudem die MRT mit zusätzlichen Schichten auf Knieebene als Alternative ohne Strahlenbelastung etabliert.

### Arthro-MRT der Hüfte

Die erweiterte Beurteilung dysplastischer Hüftgelenke erfolgt mit einer Arthro-MRT. Hochwertige Arthro-MRT beinhalten Beckensequenzen zur Erfassung von Knochen- oder Weichteilödemen (axiale/koronale flüssigkeitssensitive Sequenzen) und schnelle axiale Sequenzen zur femoralen Torsionsbestimmung. Hüftspezifische Sequenzen mit koronalen, sagittalen und axialen Sequenzen dienen der Erkennung von sekundären Labrum- und Knorpelpathologien, Ossifikationen oder Osteophyten, sowie der Bewertung der periartikulären Weichteile. Radiäre Schichten ermöglichen die Evaluation einer Cam-Deformität und einer möglichen Dezentrierung des Hüftkopfes. Zur noch differenzierteren Beurteilung des femoralen und azetabulären Knorpels oder verbesserten Erkennung von Hinweisen auf Labrumveränderungen empfehlen wir Aufnahmen unter Traktion, welche die Art der Knorpelschädigung besser darstellen.

Bei Hüftdysplasie zeigen sich im Arthro-MRT typischerweise sekundäre Hinweise auf die erhöhte Druck- und Scherbelastung oder Gelenkinstabilität.

## Sekundäre Veränderungen

**Figure 5:**
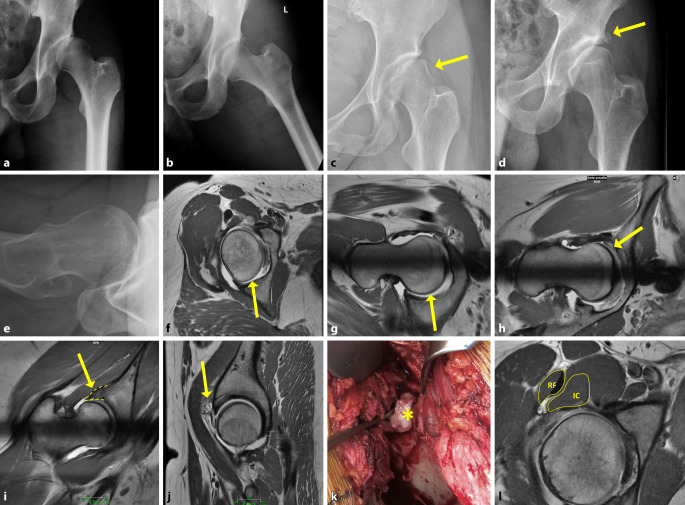

**Figure 6:**
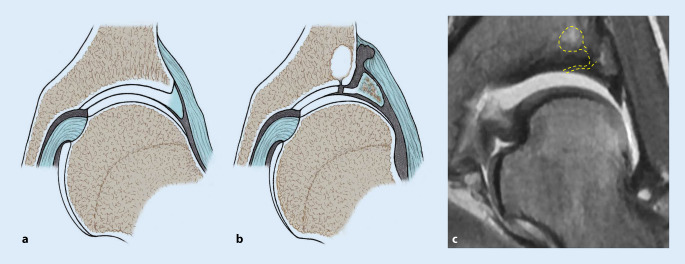

Als Folge des defizitären Containments resultieren Gelenksveränderungen und typische Läsionen, die sich im konventionellen Röntgenbild, CT oder MRT nachweisen lassen (Tab. 3; Abb. 5). Im Falle einer Borderline-Dysplasie weisen genau diese Veränderungen auf eine chronische Instabilität hin, und es wird spezifisch nach ihnen gesucht, um die Diagnose einer Hüftdysplasie zu untermauern. Hierbei ist insbesondere die dysplasietypische Inside-Out-Läsion des superolateralen Pfannenrandes [9] hervorzuheben (Abb. 6).

**Table 3:** 

Parameter	Ebene +/− Sequenz	Definition	Abb. 5
*Konventionelles Röntgen*			Abb. 5a–e
Scheinbare Gelenkspaltverschmälerung	BüS/Abduktionsaufnahme	Durch anterolaterale Subluxation des Femurkopfes scheinbar verschmälerter Gelenkspalt	Abb. 5a, b
Minimus-Delle [1]	BüS	Entrundung des superolateralen Hüftkopfanteils durch chronischen Kontakt mit der Sehne des M. gluteus minimus	*gelber Pfeil* in Abb. 5c
Hüftkopfform [28]	BüS/axiale Aufnahme	Asphärischer, elliptischer Hüftkopf mit breitem Schenkelhals und valgischer Konfiguration der Schenkelhalsachse und Bildung eines Os acetabuli (*gelber Pfeil* in Abb. 5d)	Abb. 5d, e
*Arthro-MRT*			Abb. 5f–l
Sichel-Zeichen [32]	Axiale/radiäre Schnitte mit intraartikulärem Kontrastmittel	Ungleiche Kontrastmittelverteilung um den dezentralisierten Hüftkopf mit vermehrter, sichelförmiger Ansammlung von Kontrastmittel posterior	*gelbe Pfeile* in Abb. 5f, g
Labrumschaden „inside-out-lesion“ [9]	Radiäre Schnitte	Superolaterale Pfannenrandläsion mit Abscherung von azetabulärem Knorpel und Labrum	*gelber Pfeil* in Abb. 5h
Labrumhypertrophie	Radiäre/koronare Schnitte	Breitbasiges, kompensatorisch vergrößertes und aufgelockertes Labrum (*gestrichelte Linie* entlang des Labrums in Abb. 5i)	*gelber Pfeil* in Abb. 5i
Ganglion	Sagittale/radiäre Schnitte	Paraartikuläre Ganglien im superolateralen Bereich	*gelber Pfeil* und *Asterisk* in Abb. 5j, k
Iliocapsularis-Index [4]	Axiale Schnitte	M. iliocapsularis (IC) – M. rectus femoris (RF)-Verhältnis > 1,30	*gelbe Umrandung* in Abb. 5l

*BüS* Beckenübersichtsaufnahme

## Makro- versus Mikroinstabilität

Das Hauptproblem der Hüftdysplasie stellt die Gelenkinstabilität durch die verminderte Gelenkfläche dar [21]. Deutliche Defizite des Gelenk-Containments und der Überdachung mit konsekutiver „Makroinstabilität“ lassen sich einfach erkennen und eindeutig adressieren. Eine Herausforderung stellt jedoch die Borderline-Dysplasie dar, bei denen die pathognomonischen Parameter im Grenzbereich liegen. In diesen Fällen ermöglicht das Gesamtbild aus klinischer Untersuchung und radiologischer Diagnostik die Erkennung von Zeichen einer „Mikroinstabilität“. Insbesondere die Analyse der ergänzenden radiologischen Parameter und sekundären Veränderungen, welche auf eine Gelenksinstabilität hinweisen, spielen hierbei eine zentrale Rolle. Denn entscheidend ist in der Zusammenschau aller Befunde, ob eine Hüfte stabil oder instabil ist, und weniger, ob es sich um eine „Makro-“ oder „Mikroinstabilität“ handelt. Darüber hinaus sollte zur Planung der optimalen Therapie eine Evaluation möglicherweise gleichzeitig vorliegender Hüftpathologien, wie beispielsweise femoraler Torsionsstörungen, erfolgen.

**Figure 7:**
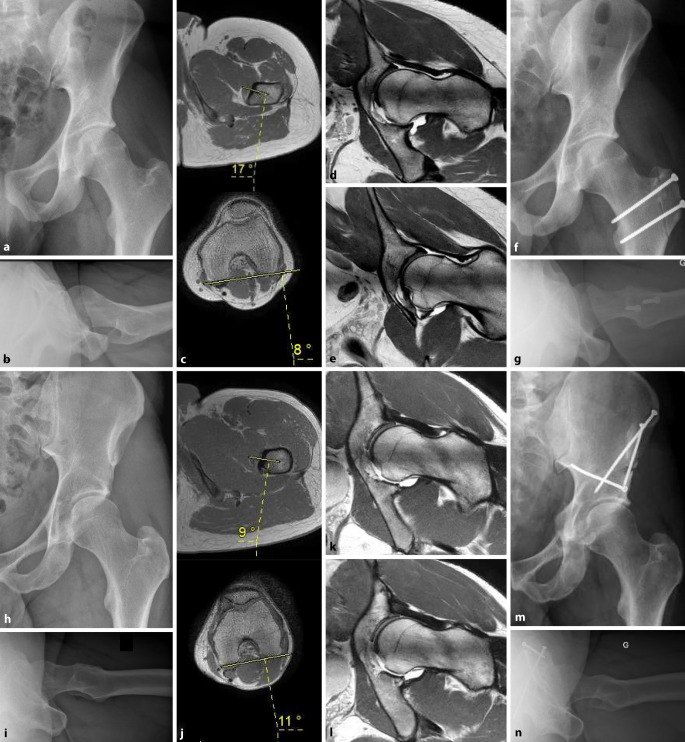

Nicht nur die Diagnose einer Hüftdysplasie, sondern auch die optimale Therapie basiert auf einer individuellen, morphologischen Analyse von Becken und Femur. Die Therapie kann sich bei nativradiologisch ähnlichem Erscheinungsbild (Abb. 7a, b, h, i) in Abhängigkeit von femoraler Torsion, azetabulärer Version und dem Vorliegen von sekundären Instabilitätszeichen (Abb. 7c, d, e, j, k, l) stark unterscheiden. Während in einem stabilen Gelenk die Impingement-Komponente beschwerdeführend ist und über eine Hüftarthroskopie oder eine chirurgische Hüftluxation adressiert wird (Abb. 7f, g), bedarf es bei einer Gelenkinstabilität einer Reorientierung des Azetabulums mittels einer PAO mit gegebenenfalls zusätzlicher knöcherner Korrektur des zeitgleich vorliegenden femoroazetabulären Konfliktes (Abb. 7m, n), um die Schmerzursache zu beheben.

Basierend auf einer vollständigen klinisch-radiologischen Analyse der Pathomorphologie sollten Indikationsstellung und chirurgische Therapie in einem Zentrum für gelenkerhaltende Hüftchirurgie durch ein Team mit langjähriger Erfahrung erfolgen.

## Fazit für die Praxis

- Ausgeprägte Dysplasien und hohe Luxation stellen mittlerweile eine Seltenheit dar und wurden durch subtilere, residuelle Pathomorphologien abgelöst.
- Die folgenden pathognomonischen Parameter erlauben einzeln oder in Kombination die Diagnose einer Hüftdysplasie: „Lateral-center-edge“(LCE)-Winkel < 22°, anteriore Hüftkopfüberdachung < 14 %, unterbrochene Shenton-Linie, insuffiziente anteriore Überdachung nach der Drittel-Regel für die azetabuläre Vorderwand und hohe Luxation.
- In Borderline-Fällen (LCE-Winkel 20–25°) gewinnen supportive und deskriptive Parameter mittels Arthro-MRT zunehmend an Bedeutung, um subtile Zeichen einer Instabilität zu detektieren.
- Es ist wichtig zu erkennen, ob eine Hüfte stabil oder instabil ist, die Unterscheidung zwischen Makro- und Mikroinstabilität ist ungenau und liefert keine Zusatzinformationen.
- Die adäquate Therapie basiert auf einer ganzheitlichen Analyse der Pathomorphologie und sollte spezialisierten Zentren für gelenkerhaltende Chirurgie vorbehalten sein.

## Abkürzungen

BüS: Beckenübersichtsaufnahme
FEAR-Index: Femoroepiphysealer azetabulärer „Roof“-Index
HW-SHA-SP: Hinterwand-Schenkelhalsachse-Schnittpunkt
LCE: „Lateral-center-edge“
MCE: „Medial-center-edge“
PAO: Periazetabuläre Osteotomie
VW-SHA-SP: Vorderwand-Schenkelhalsachse-Schnittpunkt
